# Therapeutic challenges in epidermal inclusion cysts with periocular localization: case reports

**DOI:** 10.25122/jml-2023-0539

**Published:** 2023-10

**Authors:** Anisia-Iuliana Alexa, Carmen-Ecaterina Leferman, Alin Dumitru Ciubotaru, Ioana Alexandra Sandu, Calina Anda Sandu, Camelia Margareta Bogdănici

**Affiliations:** 1 Department of Ophthalmology, “Grigore T. Popa” University of Medicine and Pharmacy, Iaşi, Romania; 2 Department of Pharmacology, “Grigore T. Popa” University of Medicine and Pharmacy, Iași, Romania; 3 Department of Neurology, “Grigore T. Popa” University of Medicine and Pharmacy Iași, Romania; 4 Department of Medical Specialties II, “Grigore T. Popa” University of Medicine and Pharmacy, Iasi, Romania

**Keywords:** epidermal inclusion cyst, oculoplastics, epidermoid cyst

## Abstract

Epidermal inclusion cysts in the periocular region are distinctive pathologies exhibiting varied clinical and radiological features, and they should be taken into consideration in the differential diagnosis of cystic lesions near the orbit. This article discusses the clinical and radiological details, along with the surgical results, of two individual cases of epidermal inclusion cysts, with different localization and without any preceding trauma, surgical history, or eyelid inflammation. In the first case, a substantial spherical structure closely connected to the tarsal plate was identified via excisional biopsy, whereas the second case involved a soft, oval tumor located at the outer right orbital corner, as determined clinically and validated through computed tomography. The histological examination showed cysts lined with a keratinized squamous layer, confirming an epidermoid cyst. The surgical removal of the cysts led to esthetically satisfactory outcomes in both cases. The particularity of the presented cases lies in the locations and considerable sizes of the tumors, which have complicated their surgical management. Such instances of epidermal inclusion cysts attached to the tarsus are rarely reported in the literature.

## INTRODUCTION

Epidermal inclusion cysts with periocular localization can occur in various parts of the orbital area and cause significant symptoms such as proptosis, enophthalmos or exophthalmos, pain, swelling, and disturbances in eye movements [1]. The risk of visual impairment is significant if the cyst affects the optic nerve or alters ocular structures.

Epidermoid cysts are uncommon lesions that may be either primary (congenital) or secondary in nature [2]. Primary epidermoid cysts typically originate during early gestation, between the third and fifth weeks, due to the displacement of epithelial elements along the neural groove or other epithelial fusion lines, whereas secondary epidermoid cysts are often a result of the posttraumatic implantation of surface epithelium. These cysts can appear at any age but are predominantly observed in younger adults.

The diagnosis relies greatly on imaging studies, such as computed tomography (CT) or magnetic resonance imaging (MRI), which aid in determining the cyst’s precise location, dimensions, and its consequent effect on surrounding orbital tissues. A critical component of the diagnosis is the differentiation of these cysts from other orbital pathologies such as dermoid cysts, teratomas, or various neoplasms. Orbit-located epidermoid cysts can derive from cutaneous, conjunctival, respiratory, or lacrimal gland epithelium [3]. Therefore, a precise histopathological examination is necessary following surgical removal, to establish a definitive diagnosis.

The primary treatment approach is surgical excision, taking into consideration factors such as cyst’s anatomical positioning and its degree of engagement with adjacent orbital structures [4]. Despite their benign nature, epidermal inclusion cysts may undergo malignant transformation in rare cases [5], underscoring the importance of accurate diagnosis and management.

We present two cases of epidermal inclusion cysts, focusing on their diverse clinical and radiological manifestations and discussing the results of surgical interventions for their removal. The first case involves a 12-year-old patient, and the second case concerns a 58-year-old male patient, each with a different localization and consequently a targeted therapeutic approach.

## CASE PRESENTATION

### Case 1

The first case involves a 12-year-old female patient with an upper eyelid lesion, first noticed by her parents when she was a few months old. Since then, it has become progressively larger and more visible, and has caused changes to the adjacent tissues over time, without a history of trauma. The girl presented a normal development for her age, with no other medical problems, no medication, no allergies, and an insignificant family history. She lived with her parents, who have been present at medical visits.

The results of the ophthalmological examination were normal, except for the examination of the adnexa, which presented a mobile, soft, tumor-like formation measuring 1.5 cm × 2.5 cm, located in the upper part of the lateral orbital rim, without sensitivity to palpation or displacement of the eyeball.

Furthermore, the native cranial and orbital CT scan revealed a well-demarcated oval lesion presenting a distinct, self-contained wall. This lesion was homogeneous in structure, except for an internal septum, and exhibited negative densities internally, being positioned at the level of the external angle of the right orbit.

**Figure 1:**
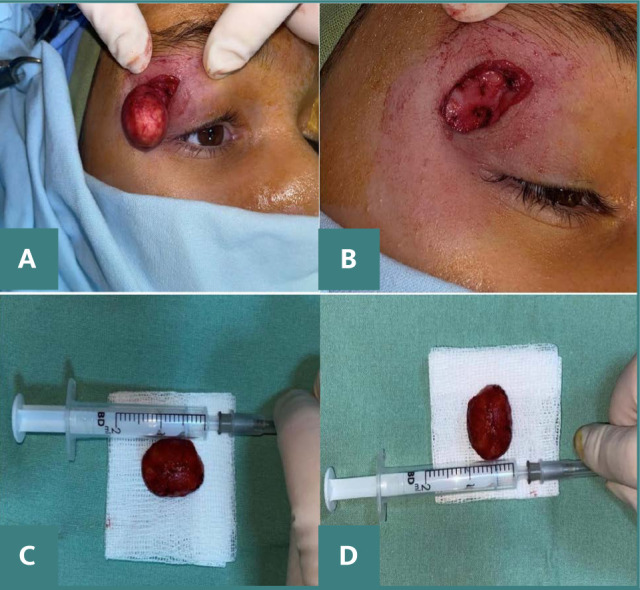
A. Epidermoid cyst removed through an upper eyelid crease incisions. B. The crater-like indentation left after the total excision of the cyst. C, D. Macroscopic aspects of the cyst removed in toto.

Considering the location and presentation of the lesion, which were indicative of a cyst, the decision was made to proceed with excision. The procedure involved performing an anterior orbitotomy, through an incision made in the crease of the upper eyelid, leading to the successful excision of the lesion (Figure 1). Maintaining the integrity of the cyst during its excision is essential, although this may not be feasible in all cases. Following the surgery, the patient received a week-long course of combined antibiotic and steroid therapy, administered both topically and systemically.

**Figure 2:**
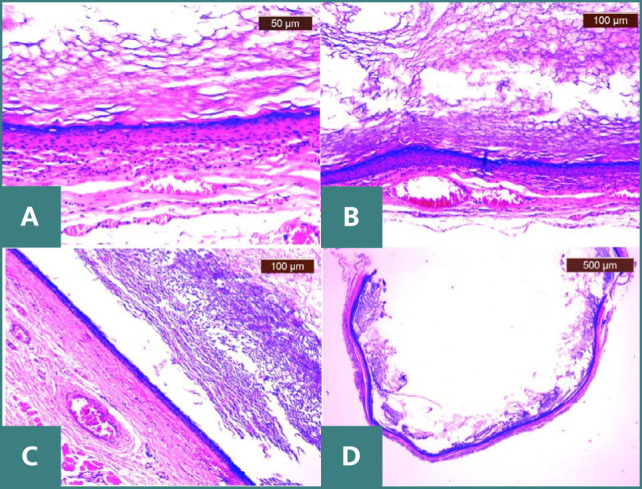
Histopathological examination showing A. inclusion cyst displaying intraluminal characteristic lamellar keratin mass (H&E, x25) B. detail of cyst wall with reduced connective support and stratified squamous epithelium with prominent granular layer (H&E, x100) C. epidermal inclusion cyst with a wall lined by stratified squamous epithelium and lamellar, loose keratin (H&E, x100) D. epidermal inclusion cyst with stratified squamous epithelium and a prominent granular layer (H&E, x200).

The histopathological evaluation confirmed the presence of an epidermal inclusion cyst (Figure 2). Microscopic examination revealed a cyst wall with diminished connective tissue support, lined by stratified squamous epithelium, which displayed a conspicuous granular layer and contained lamellar, loosely arranged keratin.

**Figure 3:**
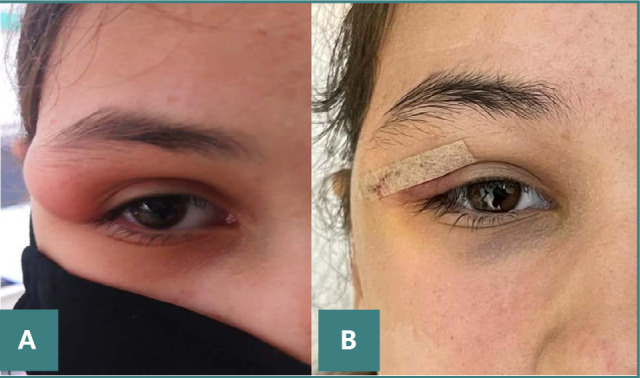
A. Preoperative photograph showing fullness of the right superior orbit. B. Third postoperative day showing favorable functional and esthetic results.

After the surgical removal of the epidermal inclusion cyst, the patient experienced a positive outcome, both functionally and esthetically (Figure 3).

### Case 2

**Figure 4:**
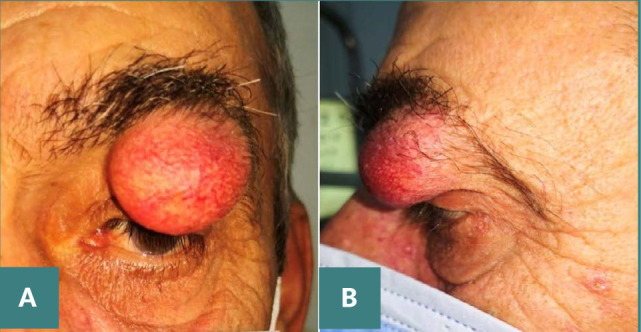
Preoperative images show a sizable mass on the upper eyelid, characterized by a round, well-defined appearance, without any indications of inflammation, causing mechanical ptosis. A. Front view. B. Side view.

The second case involves a 58-year-old patient with a large globular formation at the level of the upper eyelid, which has progressively increased in size over the last 5 years, without a history of trauma. Similar to the first case, the patient presented a normal ophthalmological examination, except for the adnexa, which revealed a large globular formation firmly attached to the tarsal plate of the upper eyelid, causing a mechanical palpebral ptosis (Figure 4).

**Figure 5:**
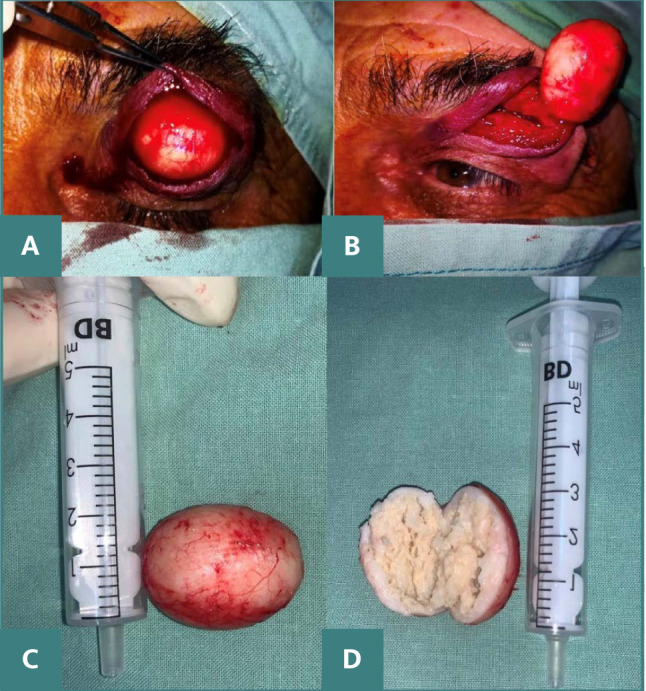
A. Intraoperative images showing a cystic mass free from the skin and subcutaneous tissue. B. Cyst removed in toto. C. Macroscopic aspect of the cyst removed in toto D. Cross-section of the excised cyst, revealing a fibrous capsule containing white-yellow, paste-like material.

The treatment involved an excisional biopsy via an incision in the crease of the upper eyelid. Surgical dissection up to the tarsus uncovered a cystic formation adhering to the tarsal plate (Figure 5). Histological analysis confirmed this to be an epidermal inclusion cyst.

The redundant skin was surgically removed, and skin closure was achieved using interrupted Proline sutures, leading to satisfactory esthetic and functional outcomes. Following the surgery, the patient received a week-long course of combined antibiotic and steroid therapy, administered both topically and systemically. The immediate postoperative course was without complications, and no recurrence of the cyst was reported during the follow-up period.

## DISCUSSION

The clinical presentation of periocularepidermoid inclusion cysts can be variable [6]. Commonly, patients present with a slow-growing, painless, and mobile mass. However, when these cysts reach a significant size or are located in cosmetically sensitive areas like the periocular region, they can lead to esthetic concerns or functional impairments, such as visual obstruction or discomfort.

In this context, the particularity of the first presented case lies in the location of the cyst, initially suggesting a potential dermoid cyst. The distinction between an epidermal inclusion cyst and a dermoid cyst is underlined by several aspects. Epidermal inclusion cysts are frequently found in regions abundant with sebaceous glands such as the face, neck, and trunk [7]. They are characterized by a keratin-filled lumen and a lining of stratified squamous epithelium, often displaying a central punctum. These cysts typically appear in adulthood and are susceptible to inflammation or infection [6]. By contrast, dermoid cysts, with a congenital basis, have both ectodermal and mesodermal origin, containing hair, sebaceous material, and occasionally teeth or bone [8]. A key diagnostic indicator is their common periocular localization, particularly near the lateral end of the eyebrow [9]. Dermoid cysts are usually observed in children as firm, non-tender nodules, less likely to become inflamed. Histologically, they exhibit a more complex structure with components from multiple germ layers [9]. The treatment for both types of cysts generally involves surgical removal, though the specific approach may differ based on the anatomical position of the cyst and potential complications [7, 10]. Therefore, precise differentiation between these cyst types is essential in clinical practice for effective management and prognosis.

Cases of epidermal inclusion cysts adherent to the tars without a history of trauma are very rarely described in the literature [11-13]. Although it is difficult to demonstrate that the tumor did not arise from an invagination of the epidermis following a trauma, we were able to exclude this type of lesion because intraoperatively we did not find adhesions between the cyst wall and the skin above it. Cysts adherent to the tarsal plate without a history of trauma, particularly ophthalmological ones, are relatively rare and can present diagnostic challenges. The tarsal plate is a dense connective tissue structure in the eyelids, providing support and shape. Typically, cysts in this region, such as chalazia and meibomiancysts, stem from blocked glands [11]. However, the presence of true cysts like epidermal inclusion cysts, generally attributed to the implantation of epidermal cells following minor trauma or surgery, indicates alternative etiological factors, possibly including congenital elements or obstructed follicles. In our case, the formation of the cyst probably took place during the embryonic development of the eyelids, through the sequestration of ectodermic tissue into that of mesodermic origin.

The clinical manifestation of these cysts, marked by symptoms from mechanical ptosis to eyelid swelling and discomfort, showcases the importance of a thorough clinical evaluation, complemented by imaging techniques and histopathological confirmation post excision [10]. This approach is vital for an accurate diagnosis and an appropriate surgical intervention, given the sensitive nature of the ocular structures involved. Moreover, due to such presentations without a trauma history being rare, a broad differential diagnosis, including both benign and malignant entities, is necessary.

Other particularities include the large dimensions of the tumor formations and the intraoperative difficulties determined by adherence to the tars. The eyelids are composed of intricate layers, including skin, muscle, connective tissue, and the tarsal plate, each with critical functions. Due to the fact that these structures are small in size, delicate in nature, and proximal one to another, surgical manipulation can prove to be challenging. Moreover, as the tarsal plate is vital for eyelid integrity and function, aggressive surgical techniques to remove an adherent cyst might damage this structure, potentially leading to complications like eyelid deformity or malposition [14]. The eyelid plays a crucial role in protecting the eye and maintaining tear film distribution. The surgical intervention must ensure that the functional integrity of the eyelid is preserved, avoiding complications like lagophthalmos. Another danger is the risk of inadvertently harming the globe, especially when dealing with deep or adherent cysts. Given the rich vascular supply of the eyelid, there is a higher risk of bleeding during surgery. Additionally, the potential for visible scarring is a concern, given the esthetic importance of the eyelid. Lastly, complete removal of the cyst is necessary to prevent recurrence [4]. However, when the cyst is adherent to the tarsal plate, achieving this without damaging surrounding tissues can be difficult. Moreover, another concern is that of an increased risk of postoperative complications such as infection, hematoma, or granuloma formation, especially if remnants of the cyst wall are left behind.

In the young patient, a transcutaneous incision along the eyelid crease was used, followed by an anterior orbitotomy for cyst removal. Meanwhile, the epidermal cyst on the tarsal plate was addressed through an excisional biopsy, necessitating detailed dissection down to the tarsus. The comprehensive surgical approach, of both the orbital and tarsal cysts, resulted in positive functional and esthetic outcomes for the patients.

## CONCLUSION

Epidermal inclusion cysts are a distinctive pathology with diverse clinical and radiological presentations, important in the differential diagnosis of periocular cystic lesions. These cysts, which may be located intraorbitally even in pediatric cases, should be considered when diagnosing dermoid cysts. Notably, epidermal inclusion cysts can also manifest in adults on the tarsal plate, even without a history of trauma. Their surgical management presents a unique challenge, aiming to ensure complete removal while preserving the cosmetic integrity of the eyelid, a particularly demanding task in instances involving large cysts or those with deep adhesions.

## Data Availability

Further data are available from the corresponding author upon reasonable request.
